# The concentration of androgen receptor and protein kinase A in male chicken following the administration of a combination of the epididymis and testicular extracts

**DOI:** 10.14202/vetworld.2020.1594-1598

**Published:** 2020-08-15

**Authors:** Muslim Akmal, Gholib Gholib, Mustafa Kamal Nasution, Sri Wahyuni, Rinidar Rinidar, Dian Masyitha, M. Aman Yaman

**Affiliations:** 1 Laboratory of Histology, Faculty of Veterinary Medicine, Universitas Syiah Kuala, Banda Aceh, Aceh, Indonesia; 2 Laboratory of Physiology, Faculty of Veterinary Medicine, Universitas Syiah Kuala, Banda Aceh, Aceh, Indonesia; 3 Department of PGMI, Faculty of Tarbiyah, STAIN Gajah Putih Takengon, Aceh Tengah, Aceh, Indonesia; 4 Laboratory of Anatomy, Faculty of Veterinary Medicine, Universitas Syiah Kuala, Banda Aceh, Aceh, Indonesia; 5 Laboratory of Pharmacology, Faculty of Veterinary Medicine, Universitas Syiah Kuala, Banda Aceh, Aceh, Indonesia; 6 Field Laboratory of Animal Sciences, Faculty of Veterinary Medicine, Universitas Syiah Kuala, Banda Aceh, Aceh, Indonesia

**Keywords:** androgen receptor, chicken, epididymis and testicular extracts, protein kinase A, spermatogenesis

## Abstract

**Background and Aim:**

Testis (T) and epididymis (E) are waste from the abattoir that is rarely used. In fact, both organs contain important chemicals needed for spermatogenesis (e.g., hormones, proteins, and other molecules). Therefore, administration of a combination of testis and epididymis (CTE) extracts may activate androgen receptors (AR) and protein kinase A (PKA) molecules that play a prominent role in spermatogenesis. We, therefore, aimed at investigating the influence of the CTE extracts on the concentration of AR and PKA in male chicken.

**Materials and Methods:**

This study used a completely randomized design with four treatment groups (K0, K1, K2, and K3) and five replications per group. K0 is a control group that received 1 mL normal saline, whereas K1, K2, and K3 are the test groups that received 1, 2, and 3 mL of CET extracts, respectively. Twenty male chickens (strain: broiler Mb 89), 3 weeks of age, weighing 500-700 g were used. We administered the injections in a 13-day period and on the 14^th^ day; we collected and processed blood samples as serum to measure the AR and PKA concentrations using commercial chicken AR and PKA enzyme-linked immunosorbent assay kits, respectively. We performed analyses by analysis of variance using SPSS 20.0.

**Results:**

The AR concentrations in K1, K2, and K3 groups increased by 4.26%, 10.97%, and 28.04%, respectively, compared to the K0 (control group). However, this increase was not significantly different between the groups (p>0.05). Moreover, the PKA concentrations increased by 2.97%, 2.60%, and 4.08% in K1, K2, and K3 groups, respectively, compared to the control group. However, this increase was not significantly different between the groups as well (p>0.05).

**Conclusion:**

The CTE extracts tended to increase the AR and PKA concentrations even though it is not significant. Therefore, it needs further study when using the CTE extracts for spermatogenesis in male chicken.

## Introduction

Nowadays, the number of slaughtered animals, including goats at abattoirs, is on the rise to meet up with meat demands from communities. This has an impact on the increase of by-products, such as testes (T) and epididymis (E). However, the use of T and E is still very limited. Unknowing to many, both organs contain hormones (follicle-stimulating hormone, luteinizing hormone, testosterone, and dihydrotestosterone) [1,2] and others molecules (cyclic AMP [cAMP]-responsive element modulator [CREM], protamine [PRM], DEFB126, CRISP1, carbonyl reductase P34H, SPAG11e, GPR64, and CD52), required for spermatogenesis [3,4]. Androgen is a steroid hormone that serves as a precursor for testosterone production (major hormone of androgen). This hormone is required in the process of spermatogenesis [5,6]. This hormone is also essential for normal expression of phenotypic expressions of the male. This includes secondary sexual characteristics, the initiation and preservation of spermatogenesis [7], phenotypic expression of normal male [8], controlling spermatogenesis [9,10], development and function of male reproductive system [11], and spermatozoa production [12]. In poultry, androgen is secreted by the testes, which later stimulates the development of hormone-sensitive tissues [13] and the growth of secondary sexual characteristics [14].

Androgen action is mediated by receptors called androgen receptors (AR) [15,16]. Signaling androgen and AR play an important role in the reproductive development of chicken [17]. Within Sertoli cells, testosterone binds to AR to activate two signaling pathways. These include the mitogen-activated protein kinase (MAPK) and calcium (Ca^2+^) pathways, which, in turn, induce a cAMP response element-binding (CREB) protein [18]. MAPK is key to various vital transduction pathway signals that regulate the proliferation, differentiation, and cell death processes in eukaryotes [19]. On the other hand, Ca^2+^ can stimulate protein kinase C (PKC) pathways, guanine nucleotide exchange factors (GEFs), or PKA that stimulates Ras (or Ras-like GTP binding protein) resulting in MAPK pathway activation [18]. PKA influences intracellular signal transduction systems to regulate proliferation, differentiation, metabolism, and other cell activities [20]. It also plays an important role as a key regulator of many cell processes [21], and in the regulation of intracellular Ca^2+^ during capacitation and spermatozoa-acrosome reaction [22]. The capacitation process begins with an enhancement in intracellular Ca^2+^, bicarbonate, and hydrogen peroxide. These collectively activate adenylyl cyclase to generate a cAMP. It then drives PKA to phosphorylate specific molecules [22]. Thus, cAMP-PKA is a pathway that influences the regulation of spermatozoa motility [23].

The previous studies reported that the administration of E-extract enhances estrogen [24], and testosterone concentrations [2], as well as spermatozoa quality of male local goat [25]. Another study by Akmal *et al*. [6] found that CET administration increased PACAP (pituitary adenylate cyclase-activating polypeptide) concentrations by 8-10%, PRM-1 concentrations by 19%, and testosterone concentrations by 47.37% in male chickens. This is due to the action of PACAP molecule in E-extract. This is in line with findings from Brubel *et al*. [26], which stated that T contains a high level of PACAP. This evidence shows that E and T are rich in PACAP as well as other molecules required for the spermatogenesis process [27].

In the current study, we aimed at investigating the effect of the administration CET extracts on the increase of AR and PKA concentrations in order to support the spermatogenesis in male chickens.

## Materials and Methods

### Ethical approval

All animals used in this experiment were approved by the Animal Ethics Committee of the Faculty of Veterinary Medicine, Universitas Syiah Kuala (Ref: 17/KEPH/IX/2018).

### Study period and location

This study was conducted from May to October 2019. Chickens were reared in a cage at teaching farm (UPT Hewan Coba), Faculty of Veterinary Medicine, Universitas Syiah Kuala.

### Epididymis and testes extraction

The extraction procedure of E and T was performed as described by Akmal *et al*. [6]. In brief, E and T of local adult male goats (bucks) aged 1.5-2 years were collected from the abattoir in Banda Aceh, Indonesia. Then, the E and T were taken to the Histology Laboratory, Faculty of Veterinary Medicine, Universitas Syiah Kuala, Banda Aceh. The samples (E and T) were then put into a container filled with water to facilitate their separation. Afterward, we cut the samples into small pieces, blended and mashed them. Furthermore, the mixture was weighed and aquadestilata was added to it (10 mL aquadestilata per gram of E and T). The solution obtained was sieved with a filter paper. Then, we centrifuged the solvent obtained at 3000 rpm for 20 min. The supernatant was filled into a 15 mL tube, stored in a freezer at −20°C before injecting them into the chickens.

### Study design

Twenty male chickens (strain: broiler Mb 89) were included, aged 3 weeks, weighing 500-700 g, and divided into four groups. We adopted a randomized sampling method to obtain four intervention groups (K0, K1, K2, and K3) with five replications per group. K0 is a control group that received 1 mL normal saline, while K1, K2, and K3 are intervention groups that received 1, 2, and 3 mL of the CET extracts, respectively. Before interventions, chickens were acclimatized for 7 days for environmental adaptation. Then, they received intramuscular injections of 1 mL of normal saline for K0 (control group), whereas K1, K2, and K3 received intramuscular injections of 1, 2, and 3 mL of the CET extract, respectively. The administrations occurred for 13 days and on the 14^th^ day, the chickens were euthanized, and necropsied to gather serum for AR and PKA measurements. During the study, the chickens were given feed and water *ad libitum*.

### Measurement of chicken AR concentrations

We measured chicken AR concentrations using a commercial chicken AR enzyme-linked immunosorbent assay (ELISA) kit (Cat. No. BZ-08051920-EB, Bioenzy) following the instruction of the manufacturer. We assayed the duplicate 50 μL AR standard into a standard well (dose range 1.25-40 ng/mL). Then, we filled the duplicate 40 μL sample (serum) into sample wells, and added 10 μL anti-AR antibody. Hereafter, we added a 50 μL streptavidin-horseradish peroxidase (HRP) to the standard and sample wells, and then thoroughly mixed. Then, we covered the microplate tightly with a microplate sealer and incubated for 60 min at 37°C. After incubation, we removed the microplate sealer before been washed 5 times with 350 μL washing buffer. Thereafter, we blotted towel paper or other absorbent material. Then, we added 50 μL of substrate A and 50 μL substrate B into each well in the microplate. The microplate was covered with a new sealer and reincubated for 15 min at 37°C in the dark. We stopped the enzyme reaction using a 50 μL stop solution. Finally, we performed absorbance measurement using a microplate reader at 450 nm. Therefore, we calculated chicken AR concentrations using the Microplate Manager-6 (MPM-6) Software (Bio-Rad Laboratories, Inc., USA).

### Measurement of chicken PKA concentrations

Chicken PKA concentrations were measured using a commercial Chicken PKA ELISA kit (Cat. No. BZ-08052920-EB, Bioenzy) following the instruction of the manufacturer. We assayed duplicate 50 μL PKA standard into the standard well (dose range 2.5-80 ng/mL). Afterward, a 40 μL duplicate sample (serum) was filled into the sample wells and then received a 10 μL anti-PKA antibody. Thereafter, we added a 50 μL streptavidin-HRP to the sample and standard wells, and mixed thoroughly. We covered the microplate with a sealer and incubated it in 60 min at 37°C. After incubation, we removed the sealer and washed it 5 times with 350 μL washing buffer. The microplate was then blotted by a towel paper or another absorbent material. We then added with 50 μL substrate A and 50 μL substrate B to each well in the microplate. Hereafter, we covered the microplate with a new sealer and reincubated it for 15 min at 37°C in the dark. After this, we stopped the enzyme reaction using a 50 μL stop solution. Finally, we determined the absorbance using a microplate reader at 450 nm. Therefore, we calculated chicken PKA concentrations using the Microplate Manager-6 (MPM-6; Bio-Rad Laboratories, Inc., USA).

### Statistical analysis

We performed data analysis by a one-way analysis of variance (ANOVA) using Statistical Package for the Social Sciences (SPSS) version 20.0 (IBM, USA). Statistical significance was set at α<0.05.

## Results

**Figure-1:**
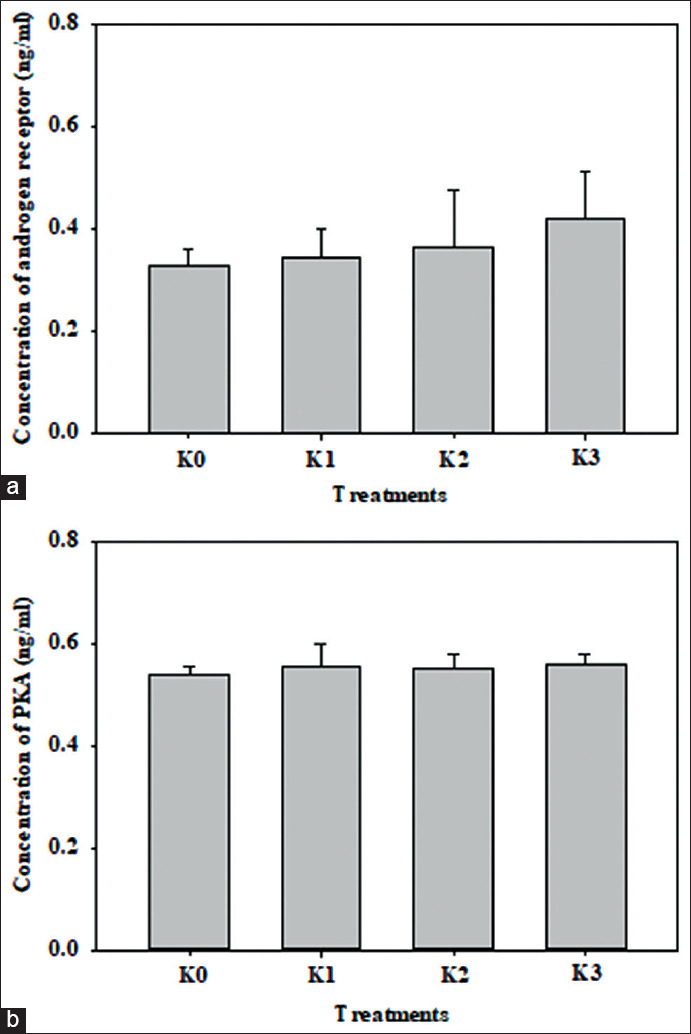
The concentration (mean ± SD) of the androgen receptor (a) and protein kinase A (b), after injected with a combination of the epididymis and testicular extracts in male chicken. There was no significant difference between groups (p>0.05). K0= chickens were injected with 1 mL normal saline, K1= chickens were injected with 1 mL combination of the epididymis and testicular extracts, K2= chickens were injected with 2 mL combination of the epididymis and testicular extracts, K3= chickens were injected with 3 mL combination epididymis and testicular extracts.

The serum AR concentrations of K0, K1, K2, and K3 were 0.32 ± 0.03 ng/mL, 0.34 ± 0.05 ng/mL, 0.36 ± 0.10 ng/mL, and 0.42 ± 0.09 ng/mL, respectively (Figure-1a). The serum AR concentrations of K1, K2, and K3 increased by 4.26%, 10.97%, and 28.04% compared to K0 (control group). However, based on ANOVA results, the serum AR concentration increase across the groups was not significantly different compared to the control group (p>0.05).

The serum PKA concentrations of K0, K1, K2, and K3 were 0.53±0.01 ng/mL, 0.55±0.04 ng/mL, 0.55±0.02 ng/mL, and 0.56±0.01 ng/mL, respectively (Figure-1b). Serum PKA concentrations in K1, K2, and K3 increased by 2.97%, 2.60%, and 4.08% compared to K0 (control group). However, based on the ANOVA results, the serum PKA concentration increase across the groups was not significantly different compared to the control group (p>0.05).

## Discussion

The current study demonstrates the effect of CET extracts (obtained from the abattoir) on the serum concentrations of AR and PKA in male chicken. Serum concentrations of AR and PKA showed no significant differences between the test and control groups. However, serum AR and PKA concentrations in test groups experienced an increase of 4.26-28.04%, and 2.97-4.08% compared to the control group.

The results of this study are closely related to the previous studies in that we can use CET extracts to support the spermatogenesis in male chickens revealed by a significant increase in the serum testosterone concentration after given CET-extracts [6]. We believe that the increase of the serum AR concentrations (4.26-28.04%) and PKA (2.97-4.08%) in this study is related to testosterone concentrations stimulated by signaling androgen in chicken testicular tissue [6]. Androgens regulate spermatogenesis [28], while signaling androgens play a very important role in the process of spermatogenesis [10]. The action of androgens (testosterone and dihydrotestosterone) in the male reproductive system is mediated by AR [29]. Signaling androgen hormone(s) is (are) indispensable for the growth and functioning of the adult male reproductive system [30], whereas signaling AR play an important role in maintaining spermatogonial numbers, blood-testicular barrier integrity, completion of meiotic division, spermatid adhesion, and the spermiation process [11].

The previous studies revealed that the testosterone-AR complex affects two molecular pathways; the MAP kinase and Ca^2+^. Testosterone activates Ca^2+^ influx into the Sertoli cells causing calmodulin to translocate to the nucleus and slowly phosphorylate CREB in about 60 s. Slowly, Ca^2+^ also triggers the PKC, GEFs, or PKA, to stimulate Ras or Ras-like GTP binding protein needed to begin the MAP kinase pathway. The MAP kinase and Ca^2+^ pathways can induce phosphorylation of CREB and CREB-mediated gene expression in the Sertoli cells [18]. Other studies reveal that testosterone is also able to influence the expression of CREM in testicular tissue [31]. Therefore, we suspect that the administration of CET-extracts provokes the activation of cAMP secretion in the sample [32], which further induces Leydig cells to carry on with steroidogenesis [33]. Our previous study revealed that the administration of E-extracts raises the estrogen secretion [24], testosterone secretion [2], and the quality of male local goat spermatozoa [25]; meanwhile, the PACAP concentrations (8-10%) and PRM-1 concentrations (6-19%) significantly raises the testosterone level [6].

The testosterone-AR complex in the cytoplasm of Sertoli cells influences adenylate cyclase to stimulate cAMP, which, in turn, drives PKA to open Ca^2+^ channels on the outside of the acrosomal membrane thereby leading to a slight increase in intracellular Ca^2+^ [22]. This increase activates the phospholipase Cγ paired with the second receptor of tyrosine kinase. This becomes a significant factor for the fusion of membranes and reaction of spermatozoon acrosome [22]. The PKA directly regulates the phosphorylation of CREB and CREM molecules and also induces the activating transcription factor-1 (ATF-1) [34]. CREB and ATF-1 influence some important signals in preserving the viability of cells during the growth of the early embryonic stage [35]. Conversely, CREB and CREM are the molecular regulators of all stages in spermatogenesis [36]. Furthermore, the Ca^2+^ pathway and cAMP-dependent PKA pathway are responsible for spermatozoa motility [22] and male fertility [37].

A possible limitation of our study was its small sample size. The serum concentrations of PKA and AR increased in the test groups. However, this increase was not significant. Therefore, we recommend studies with larger sample sizes, to obtain a reliable effect of the CET administration on serum PKA and AR concentrations.

Finally, the molecular signaling pathway mechanisms in spermatogenesis are very complex and require more explorations in future studies. Moreover, recent evidences depict that the administration of CET extracts supports the process of spermatogenesis in the male chicken.

## Conclusion

The CET extracts tended to increase the AR and PKA concentrations even though it is not significant. Therefore, it needs further study when using CET extracts for spermatogenesis in male chickens.

## Authors’ Contributions

MA and GG designed the experiments. MA, MKN, and SW performed the experiments. GG measured the AR and PKA concentration. DM and MAY provided logistical support for the experiments. MA and RR analyzed the data. MA and GG wrote the manuscript. All authors read and approved the final manuscript.

## Competing Interests

The authors declare that they have no competing interests.

## Publisher’s Note

Veterinary World remains neutral with regard to jurisdictional claims in published institutional affiliation.
